# An all-digital RF transmitter architecture based on jitter-enhanced PWM for flexible frequency applications

**DOI:** 10.1038/s41598-025-92301-4

**Published:** 2025-03-19

**Authors:** Biao Long, Wei Chen, Zhuhua Hu, Yong Bai, Dake Liu, Han Liu

**Affiliations:** 1 State Key Laboratory of Marine Resource Utilization in South China Sea, Hainan University, 58 Renmin Avenue, Haikou, 570228 Hainan China; 2 College of Information and Communication, National University of Defense Technology, 45 Jiefang Park Road, Wuhan, 430010 Hubei China

**Keywords:** Jitter-enhanced, PWM hardware architecture, Flexible frequency, Low PWM frequency, Electrical and electronic engineering, Information technology

## Abstract

We propose a novel flexible jitter-enhanced pulse width modulation(PWM) hardware architecture, which aims to improve the modulation resolution while reducing the requirement of PWM frequency. The architecture creatively distributes the jitter effect uniformly to every radio frequency(RF) cycle corresponding to the same baseband cycle by controllable jitter, which provides a new modulation resolution growth method in the time domain. The architecture provides flexibility to adapt to different basebands and radio frequencies. By using jitter, the modulation resolution of this paper can improve 5.32 bit at most, and the EVM performance can reach -41.21dB. The architecture proposed in this paper can use lower PWM frequency of 400MHz, which greatly reduces the frequency requirement of switching power amplifier in RF transmitting system. The jitter method proposed in this paper significantly improves the low modulation resolution of the traditional PWM all-digital RF transmitter and greatly changes the efficiency of all digital transmitters in the wireless communication system.

## Introduction

The core of the RF communication system is the RF transmitter, which is the architecture responsible for encoding information into RF signals. A key to this process is quantization modulation, which is a technique for converting continuous signals into discrete levels. Efficient quantization modulation is very important for high accuracy and low power consumption in the transmission process. At present, the mainstream quantization modulation techniques are Delta-Sigma modulation (DSM)^1–3^ and pulse width modulation (PWM).

DSM technology is known for its ability to provide high resolution and dynamic range. For example, a multimode 8 to 4 bits delta sigma digital-to-analog converter (DAC) is proposed in reference^4^, and a FIR filter is used in the design to reduce error and SNDR deterioration. A team designed a high over-sampling rate (OSR) pulse transmitter for FPGA applications^5^ and explored the implementation of DSM parallel topology and technology. The work of reference^6^ can transmit 10MHz 256-QAM signals in the range from 0.1GHz to 7.62GHz, and the accuracy conforms to the 3GPP specification. Although DSM modulation technology has many advantages, in view of the limitations of DSM modulation principle, a very high oversampling rate^7^ is needed to meet the in-band linearity and eliminate noise as much as possible. Due to the shortcomings such as high hardware requirements and high clock cost, there are still many challenges in applying DSM modulation technology to digital transmitters.

Compared with DSM, PWM has more advantages in complexity, hardware cost and low clock requirements. Because of its simplicity , PWM has been gradually used in all-digital transmitter^8–10^. For example, the reference^11^ proposed a design based on FPGA, which uses carrier-based PWM technology to synthesize different output frequencies. The reference^12^ uses 2140 MHz PWM for small cells to modulate 64-QAM LTE DL signals with 10 MHz bandwidth. A baseband model for uniformly sampling RF-PWM is proposed in reference^13^, and suggestions are given to use the harmonics of PWM to form carrier signals. In addition, multilevel PWM technology is also widely used. A topology of long range navigation(Loran) PWM transmitter using switching amplifier and its basic construction module is proposed in reference^14^, which can be easily implemented by traditional hardware. The reference^15^ proposes a three-level direct digital RF modulation algorithm for all-digital transmitter based on mapping, which has the advantages of coding efficiency and flexibility. A five-level RF-PWM scheme is proposed in reference^16^, which can significantly relax the filtering requirements and improve the broadband performance of all-digital transmitters. With the gradual increase of the level, the performance and efficiency of the transmitter are improved, resulting in a sudden increase in the complexity of the transmitter architecture. However, the traditional PWM technology still has the problem of insufficient modulation resolution in the high-order modulation mode, which forces the PWM time resolution to reach more than 10GHZ.

With the demand of social life for communication, the power of radio frequency signal is also increasing. To ensure the amplification linearity of the linear power amplifier, the working range of the linear power amplifier must return to the power back-off area^17,18^. In the application design of modern digital transmitter, switching power amplifier has gradually become a better choice because of the problem of power back-off. As the core device of amplifying RF signal, switching power amplifier has the advantages of high output efficiency and low power consumption. Compared with the linear power amplifier (the driving signal is a continuous RF modulation signal in time domain), the driving signal of the switching power amplifier is a modulated level signal with pulse width. The output efficiency of the switching power amplifier can reach more than 60%^19–21^. PWM signal is usually suitable for switching power amplifier, because pulse signal can effectively control switching power amplifier^22,23^. If the problem of modulation resolution is solved by increasing the PWM frequency, the cost of switching power amplifier used in RF transmitters will increase sharply, which increases the construction cost of RF transmitters.

For this reason, a new architecture based on jitter-enhanced method is proposed to improve the modulation resolution of PWM. Jitter is evenly distributed in every RF cycle corresponding to the same baseband period, providing a new modulation resolution increase in the time domain. The complexity of the control circuit is simplified by look-up table (LUT). The jitter-enhanced PWM hardware architecture proposed in this paper can flexibly adapt to different baseband and RF frequencies, and can significantly reduce the PWM frequency while achieving excellent EVM performance. This innovative architecture helps to reduce the frequency requirements and complexity of the switching power amplifier structure in the RF transmitting system of small cells. Finally, this paper gives the comparison and analysis of this scheme with others. The jitter method proposed in this paper significantly improves the low modulation resolution of traditional PWM all-digital RF transmitters and greatly changes the efficiency of all digital transmitters in wireless communication systems.

## Principle of jitter-enhanced PWM algorithm

The jitter-enhanced algorithm used in this paper was first proposed in reference^24^, on the basis of which research and innovation are carried out. We will briefly review the algorithm and put forward our own new point.

RF modulation is based on RF= A cos(2$\pi$ $f_{RF}$t + $\phi$), where $f_{RF}$ is the RF carrier frequency. In traditional PWM modulation, the resolution of PWM is limited. If the phase and amplitude modulation resolution are respectively $\Delta \phi$ and $\Delta A$, $f_{BB}$ is the baseband frequency, and $f_{PWM}$ represents the reciprocal of the PWM time resolution, then the quantization resolution of the phase is $\Delta \phi$=$f_{BB}$/$f_{PWM}$, and the quantization resolution of the amplitude is $\Delta A$= $f_{RF}$/2$f_{PWM}$ (only half of the RF period can be used for amplitude PWM). After the amplitude A and phase $\phi$ are quantized, the RF signal becomes RF =$A_{1}$cos($\omega _{0}$t+$\phi 1$), where A1 and $\phi 1$ are quantized values of A and $\phi$, respectively. When jitter is introduced, the expression of the RF signal is:

1 $$\begin{aligned} RF_{jitter} = (A_{1}+A_{jitter})cos[\omega _{0}t+(\phi _{1}+\phi _{jitter})] \end{aligned}$$

$A_{jitter}$ and $\phi _{jitter}$ represent the accuracy improved by amplitude and phase jitter, respectively. Next, we will briefly introduce how to obtain $A_{jitter}$ and $\phi _{jitter}$ to make the resulting jitter RF signal close to the result before quantization.

Taking phase jitter as an example, suppose that two phase values of $\phi _{1}$ and $\phi _{2}$ are used to generate two RF signals RF1 and RF2 with the same amplitude but different phases.$RF1= A cos(2\pi f_{RF} t + \phi 1),RF2= A cos(2\pi f_{RF} t + \phi 2)$. $\phi _{1}$ and $\phi _{2}$ are two values of adjacent quantization intervals, with $\phi _{2}=\phi _{1}+\Delta \phi$.The ratio of jitter is calculated based on the distance between the actual value of the phase and the two quantized values adjacent to the value. The signals RF1 and RF2 are selected according to the jitter ratio. If the jitter times of RF1 and RF2 are defined as k1 and k2, then k1+k2=NRF. After the receiver baseband filter, the output jitter phase value $\phi _{jitter}$ = [k1*$\phi _{1}$ + k2*$\phi _{2}$]/(k1+k2). The final RF signal is a mixture of two RF signals. The analysis process of jitter amplitude modulation is similar to that of jitter phase modulation.

**Fig. 1:**
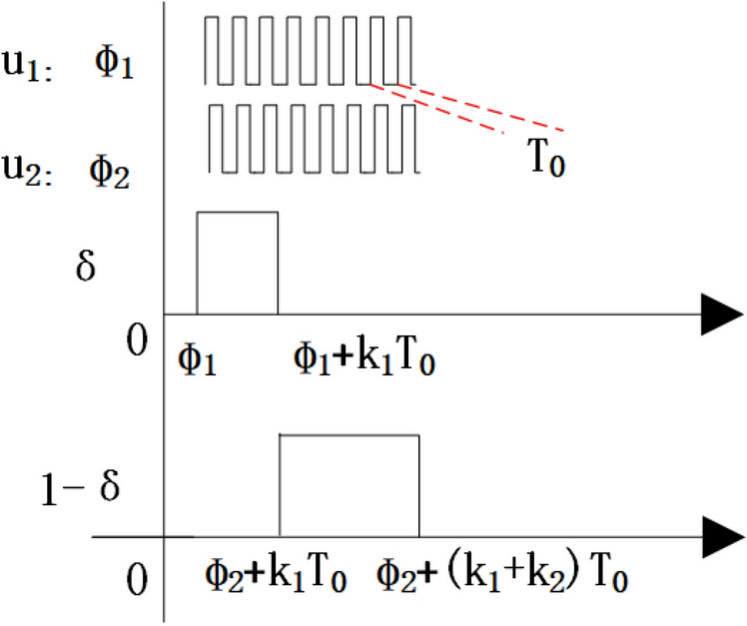
Two jitter PWM signals used in this paper.

In fact, the RF signals used in this paper are two different square wave signals with delay as jitter inputs, as shown in Fig. 1. Among them, the RF period is $\hbox {T}_{0}$ and the baseband period is T. The duty cycle of the two PWM jitter signals is $\hbox {D}_{1}$ and $\hbox {D}_{2}$ respectively, and the phase delay lines are $\phi _{1}$ and $\phi _{2}$ respectively. Suppose that in the same baseband cycle, the first K1 RF cycle transmits u1, and the last K2 RF cycle transmits u2. So two PWM signals for jitter such as Eqs. (2), (3) can be obtained.

2 $$\begin{aligned} u_{1}= & \frac{D_{1}}{2}+\frac{2}{\pi } \sum _{n=1}^{\infty } \frac{1}{n}\left( \sin \left( n \pi D_{1}\right) \cdot \cos \left( \frac{2 n \pi }{T_{0}}\left( t-\phi _{1}\right) -n \pi D_{1}\right) \right) \end{aligned}$$

3 $$\begin{aligned} u_{2}= & \frac{D_{2}}{2}+\frac{2}{\pi } \sum _{n=1}^{\infty } \frac{1}{n}\left( \sin \left( n \pi D_{2}\right) \cdot \cos \left( \frac{2 n \pi }{T_{0}}\left( t-\phi _{2}\right) -n \pi D_{2}\right) \right) \end{aligned}$$

We define a square wave to represent the alternating switch of K1 and K2, and(K1+K2)$\hbox {T}_{0}$=T, to simulate the jitter behavior of alternating transmission. In this way, the switching function such as the Eq. (4) can be obtained. When the two PWM square waves go through jitter transmission, the duty cycle and delay can be modulated more accurately. Convert Eq. (4) into a Fourier series to obtain Eq. (5).

4 $$\begin{aligned} \delta= & {\left\{ \begin{array}{ll}1,N\big (K_1+K_2\big )T_0+\Phi _1<t\le \\ N\big (K_1+K_2\big )T+K_1T_0+\Phi _1\\ 0,N\big (K_1+K_2\big )T_0+K_1T_0+\Phi _2<t\le \\ N\big (K_1+K_2\big )T_0+\big (K_1+K_2\big )T_0+\Phi _2\end{array}\right. },N=1,2,3,\ldots \end{aligned}$$

5 $$\begin{aligned} \delta= & \frac{k_{1}}{k_{1}+k_{2}}+\sum _{n=1}^{\infty } \frac{2 \sin \frac{k_{1} n \pi }{k_{1}+k_{2}}}{n \pi } \cos \left( \frac{k_{1} \pi n}{k_{1}+k_{2}}-\frac{2 n \pi }{\left( k_{1}+k_{2}\right) T_{0}}\left( t-\Phi _{1}\right) \right) , n=1,2,3, \ldots \end{aligned}$$

According to the above definition, the transmitted signal **u** can be expressed as Eq. (6).

6 $$\begin{aligned} u=u_{1} \delta +u_{2}(1-\delta ) \end{aligned}$$

To simplify the discussion, we only consider the DC component and the fundamental wave (n=1) in u1, u2, and $\delta$. Take the fundamental wave for u1 and u2 and the DC component for $\delta$, then Eq. (6) can obtain Eq. (7), where X represents irrelevant terms for other frequencies and needs to be filtered out with a band-pass filter.

7 $$\begin{aligned} \begin{aligned}&u=u_{1}\delta +u_{2}(1-\delta ) \\&=X+\frac{2A}{\pi (k_{1}+k_{2})}\sqrt{k_{1}^{2}\sin ^{2}(\pi D_{1})+k_{2}^{2}\sin ^{2}(\pi D_{2})+2k_{1}k_{2}\sin (\pi D_{1})\sin (\pi D_{2})\cdot \cos (\frac{2\pi }{T_{0}}(\Phi _{2}-\Phi _{1})-\pi (D_{2}-D_{1}))} \\&\cdot \cos (\frac{2\pi }{T_{0}}t+\tan ^{-1}\frac{-k_{1}\sin (\pi D_{1})\sin (\frac{2\pi }{T_{0}}\Phi _{1}+\pi D_{1})-k_{2}\sin (\pi D_{2})\sin (\frac{2\pi }{T_{0}}\Phi _{2}+\pi D_{2})}{k_{1}\sin (\pi D_{1})\cos (\frac{2\pi }{T_{0}}\Phi _{1}+\pi D_{1})+k_{2}\sin (\pi D_{2})\cos (\frac{2\pi }{T_{0}}\Phi _{2}+\pi D_{2})}) \end{aligned} \end{aligned}$$

Because the accuracy of amplitude jitter and phase jitter is actually the quantization accuracy of jitter, the value obtained by subtracting the two jitter values is very small, so the amplitude $A_{out}$ after jitter can be approximated as Eq. (8).

8 $$\begin{aligned} A_{out}\approx \frac{2A}{\pi (k_1+k_2)}\left[ k_1\sin (\pi D_1)+k_2\sin (\pi D_2)\right] \end{aligned}$$

It can be seen from Eq. (8) that the amplitude after synthesis is related to the sin value and jitter ratio of the pulse width and amplitude signal before synthesis.

If only phase jitter is considered, let $D1=D2=D$, $\phi _{2}=\phi _{1}+\Delta \phi$, then the phase out $\phi _{out}$ derivation process after jitter is shown in Eq. (9). It can be seen from Eq. (9) that the synthesized phase is related to the jitter ratio. As k2 increases, the number of right jitter increases, and the delay of the new phase delay line will increase. The improved jitter-enhanced PWM algorithm is shown in Algorithm 1.

9 $$\begin{aligned} \begin{aligned}&\Phi _{out}=\tan ^{-1}\frac{-k_{1}\sin (\pi D)\sin (\frac{2\pi }{T_{0}}\Phi _{1}+\pi D)-k_{2}\sin (\pi D)\sin (\frac{2\pi }{T_{0}}\Phi _{2}+\pi D)}{k_{1}\sin (\pi D)\cos (\frac{2\pi }{T_{0}}\Phi _{1}+\pi D)+k_{2}\sin (\pi D)\cos (\frac{2\pi }{T_{0}}\Phi _{2}+\pi D)} \\&=\tan ^{-1}(-\frac{k_{1}\sin (\frac{2\pi }{T_{0}}\Phi _{1}+\pi D)+k_{2}\sin (\frac{2\pi }{T_{0}}\Phi _{2}+\pi D)}{k_{1}\cos (\frac{2\pi }{T_{0}}\Phi _{1}+\pi D)+k_{2}\cos (\frac{2\pi }{T_{0}}\Phi _{2}+\pi D)}) \\&\approx \tan ^{-1}(-\frac{k_{1}\sin (\frac{2\pi }{T_{0}}\Phi _{1}+\pi D)+k_{2}\sin (\frac{2\pi }{T_{0}}\Phi _{1}+\pi D)+k_{2}\cos (\frac{2\pi }{T_{0}}\Phi _{1}+\pi D)\Delta \Phi }{k_{1}\cos (\frac{2\pi }{T_{0}}\Phi _{1}+\pi D)+k_{2}\cos (\frac{2\pi }{T_{0}}\Phi _{1}+\pi D)-k_{2}\sin (\frac{2\pi }{T_{0}}\Phi _{1}+\pi D)\Delta \Phi }) \\&=\tan ^{-1}(-\frac{(k_{1}+k_{2})\sin (\frac{2\pi }{T_{0}}\Phi _{1}+\pi D)+k_{2}\cos (\frac{2\pi }{T_{0}}\Phi _{1}+\pi D)\Delta \Phi }{(k_{1}+k_{2})\cos (\frac{2\pi }{T_{0}}\Phi _{1}+\pi D)-k_{2}\sin (\frac{2\pi }{T_{0}}\Phi _{1}+\pi D)\Delta \Phi }) \\&\approx \tan ^{-1}(-\tan (\frac{2\pi }{T_{0}}\Phi _{1}+\pi D)\frac{k_{2}\Delta \Phi }{k_{1}+k_{2}}) \\&=-(\frac{2\pi }{T_{0}}\Phi _{1}+\pi D)\frac{k_{2}\Delta \Phi }{k_{1}+k_{2}} \end{aligned} \end{aligned}$$

**Algorithm 1:**
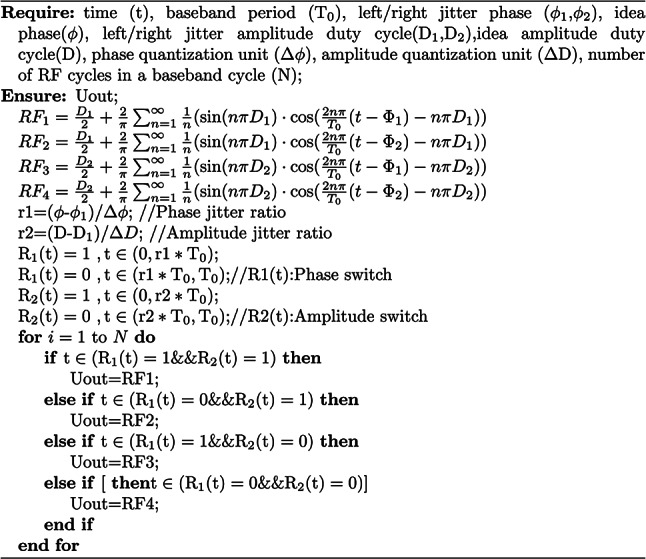
Pseudocode of improved jitter-enhanced PWM.

Jitter can provide multiple opportunities for accurate analysis in a baseband cycle, which is the basic principle that jitter can improve accuracy in hardware. The reason why the jitter can be analyzed by the receiver is that there is a baseband filter in the receiver. The baseband filter itself is an integrator and the frequency response of the filter is usually determined by the time constant^25^. The model without jitter only brings resolution in pulse width, but jitter brings new resolution opportunities in time domain. Suppose N times of jitter occurs in a baseband cycle, which is the change of N times energy distribution ratio in time. The baseband filter integrates the jitter effect, converts it into the energy distribution on the baseband, and a more accurate phase/amplitude in the baseband period is obtained.

The baseband low-pass filter refines the energy distribution to an accurate value by integrating signal. In the reference^26^ study of a three-phase PWM rectifier, a low-pass filter is used instead of an integrator to integrate the converter voltage to calculate the virtual magnetic flux. In this paper, the low-pass filter of the receiver can be skillfully used as an integrator to integrate the jitter signal at the transmitter, to analyze the resolution enhancement effect brought by jitter. This is an ingenious innovation of the hardware architecture proposed in this paper.

## System model

### Jitter-enhanced all-digital transmitter architecture

**Fig. 2:**
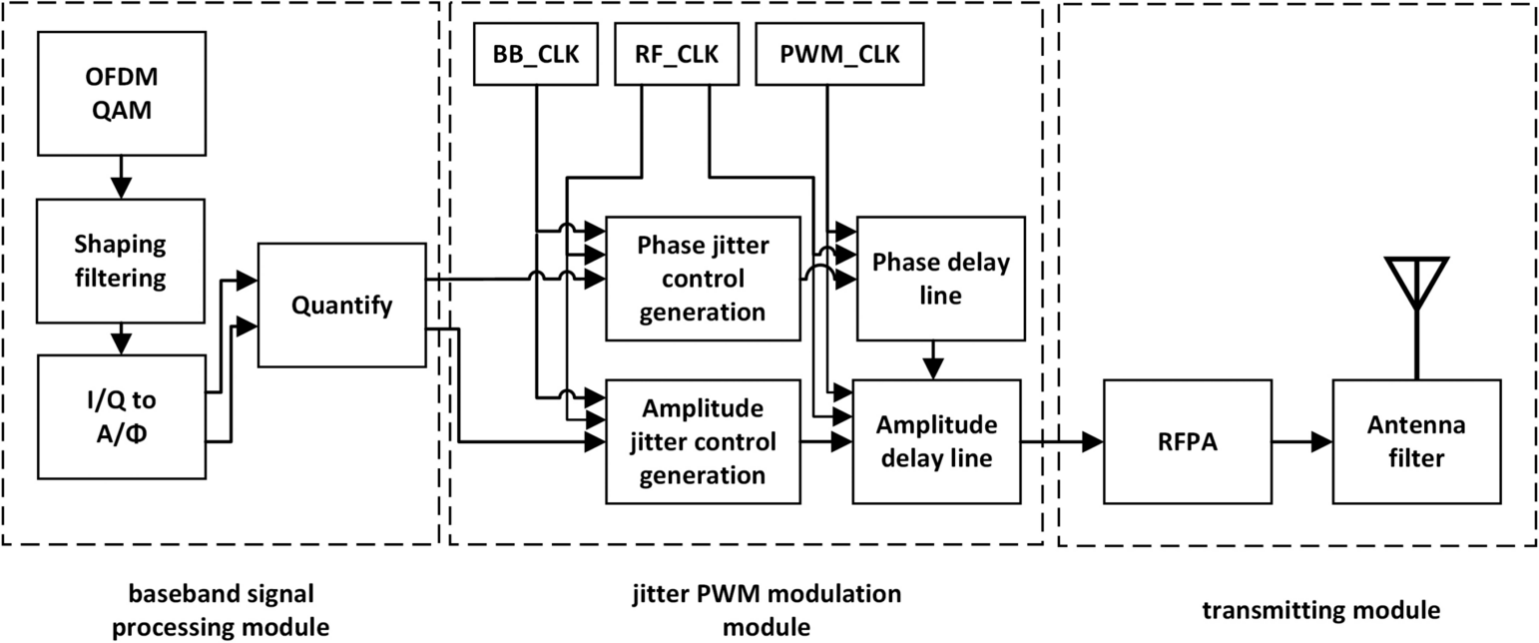
The architecture of jitter-enhanced full-digital PWM transmitters.

The structure of a typical jitter-enhanced all-digital PWM transmitter is shown in Fig. 2, which mainly consists of three parts: baseband signal processing module, jitter PWM modulation module and transmitting module. The main work of this paper focuses on the jitter PWM modulation module.

First of all, the baseband signal processing module of the all-digital transmitter converts the I/Q signal into the form of amplitude and phase^27–29^, and calculates the control signal required for subsequent jitter in the process of quantization. These complex calculations can be done on the server in the launch system. To solve the problem of phase or amplitude jump when switching between different baseband periods, a shaping filter is used after IQ data modulation to eliminate high-frequency components and ensure that the RF signals on the antenna are smooth and continuous. Then the data is rounded, sampled and mapped to the control signal at the entrance of the delay line by the quantization LUT. The jitter PWM module is designed based on the delay line architecture and can be completed using FPGA or ASIC. Through the jitter control unit of the delay line, the left output and right output of the filp-flop can be controlled, and the baseband signal is modulated into RF signal after jitter processing. For the modulated signal processed by delay line, the relative position and pulse width of the pulse contain the phase and amplitude information respectively. In the transmitting module, the full swing PWM waveform is first amplified by the switching power amplifier, and then filtered by the RF filter. In this way, the PWM waveform is converted into an amplitude modulated RF antenna signal and transmitted.

### Jitter-enhanced PWM delay line architecture

**Fig. 3:**
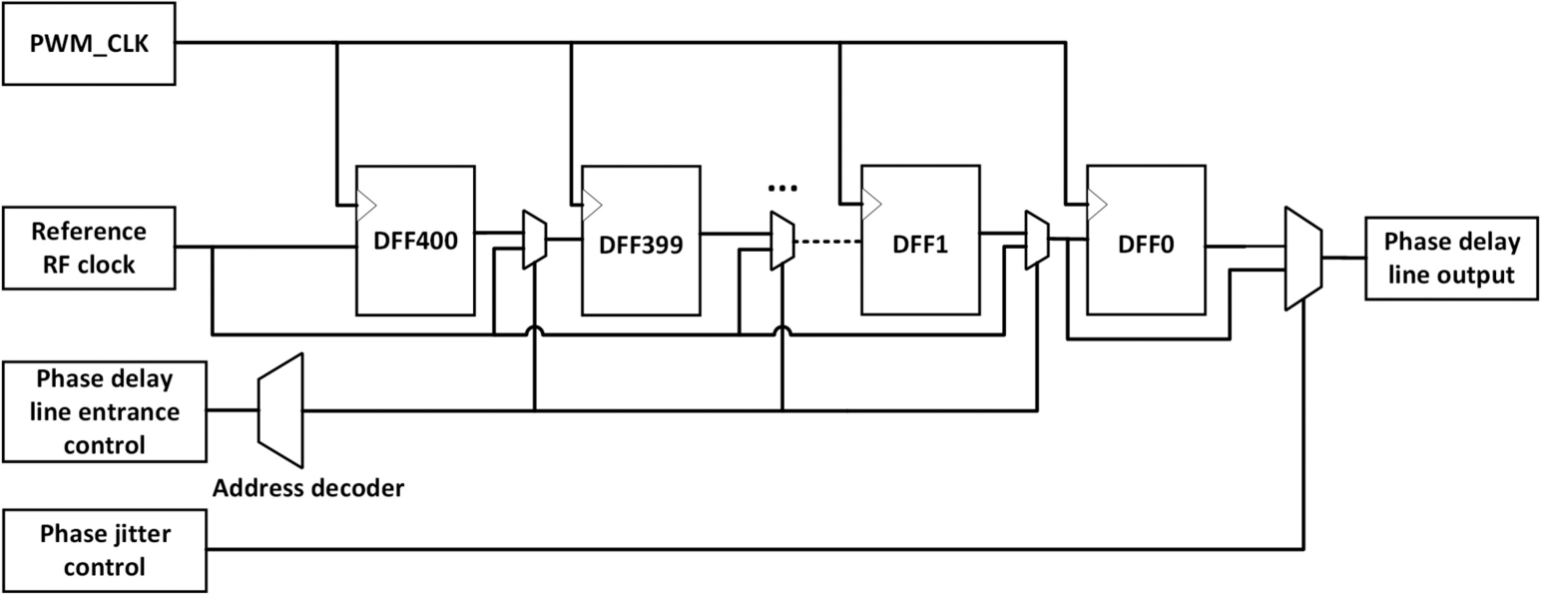
Phase delay line architecture.

**Fig. 4:**
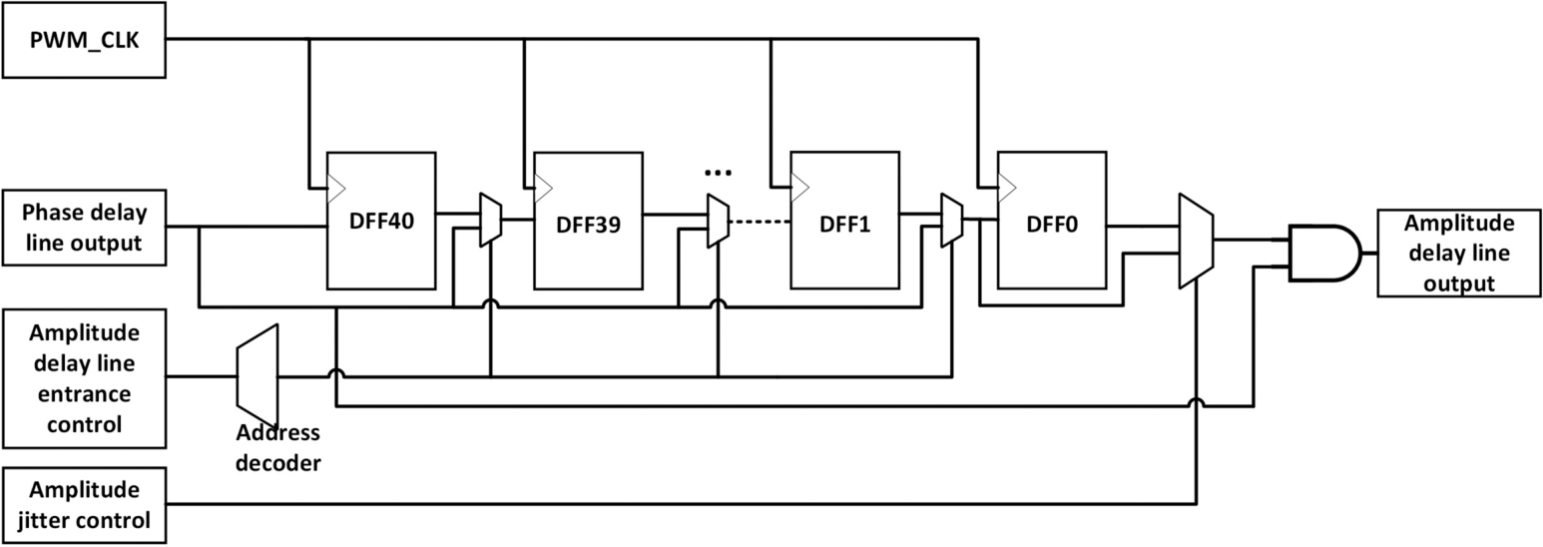
Amplitude delay line architecture.

This section mainly describes the architecture of the improved jitter delay line. The baseband phase delay line architecture and radio frequency amplitude delay line architecture are shown in Figs. 3 and 4.

**Fig. 5:**
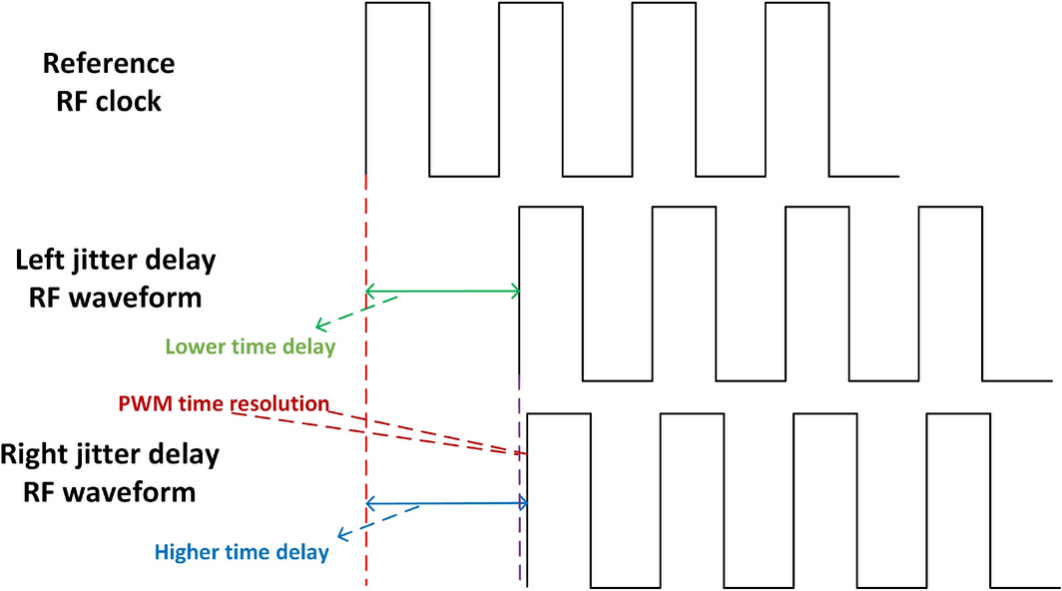
Phase jitter PWM waveform.

The phase, amplitude information and jitter control signals generated by the baseband signal processing module first control the phase delay line architecture. The phase delay line reflects the delay of the signal in the baseband period, which requires the overall right delay of all RF square wave signals in a baseband period. The waveform diagram of the phase jitter is shown in Fig. 5. Compared with the reference RF clock, the left jitter waveform means a lower time delay and the right jitter waveform means a higher time delay. The left and right jitter of phase modulation represent two adjacent quantization intervals of phase quantization, and the difference of their time delay corresponds to the PWM time resolution. The control logic of the phase delay line is controlled by the phase information, which determines the position of the RF clock at the entrance address of the input delay line. The phase jitter control logic controls whether the original RF square wave signal is output in the DFF1 (left jitter) or in the DFF0 (right jitter), as shown in Fig. 3. In this way, the delay of each RF square wave can be controlled with a slight left and right jitter to bring about a better phase delay modulation.

**Fig. 6:**
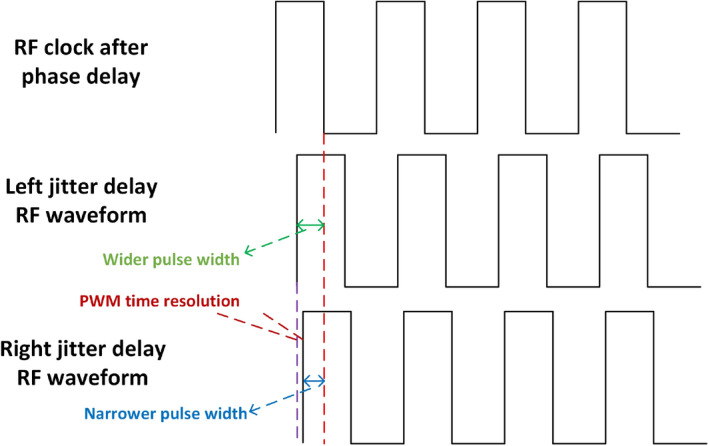
Amplitude jitter PWM waveform.

After the phase modulation of the phase delay line is completed, the delayed RF clock signal will be the input of the amplitude delay line architecture. The more delay through the amplitude delay line, the narrower pulse width obtained by the ”and” operation between the delayed RF signal and the RF signal output from the phase delay line. The waveform diagram of the amplitude jitter is shown in Fig. 6. The reference signal of the amplitude delay line is the RF clock signal modulated by the phase delay line. Compared with the reference signal, the left jitter waveform means a wider pulse width, and the right jitter waveform means a narrower pulse width. The left and right jitter of amplitude modulation represent two adjacent quantization intervals of amplitude quantization, and the difference of pulse width corresponds to the PWM time resolution. Similar to the phase delay line, the amplitude jitter control logic controls whether the reference RF waveform after phase modulation is output in the DFF1 (left jitter) or in the DFF0 (right jitter), as shown in Fig. 4. So the pulse width of each RF square wave can be controlled with a slight left and right jitter to bring about a finer amplitude delay modulation.

The phase delay line uses a PWM clock with a maximum frequency of 400 MHz to provide a resolution frequency, delaying the baseband signal by up to 4 MHz. According to the ratio of PWM frequency to baseband frequency, we provide at most 400 D flip-flops for phase delay modulation. If the user’s baseband/RF frequency changes, the flexible selection of frequency can be realized by controlling the number of D flip-flops used. According to the number of D filp-flops used in the phase delay line, we can flexibly choose the baseband frequency and the PWM frequency. At the same time, according to the user’s RF frequency, the number of D flip-flops is controlled to realize the flexible selection of RF frequency. Flexible baseband / RF frequency selection is the innovation of our proposed architecture.

### Jitter PWM control flow

**Fig. 7:**
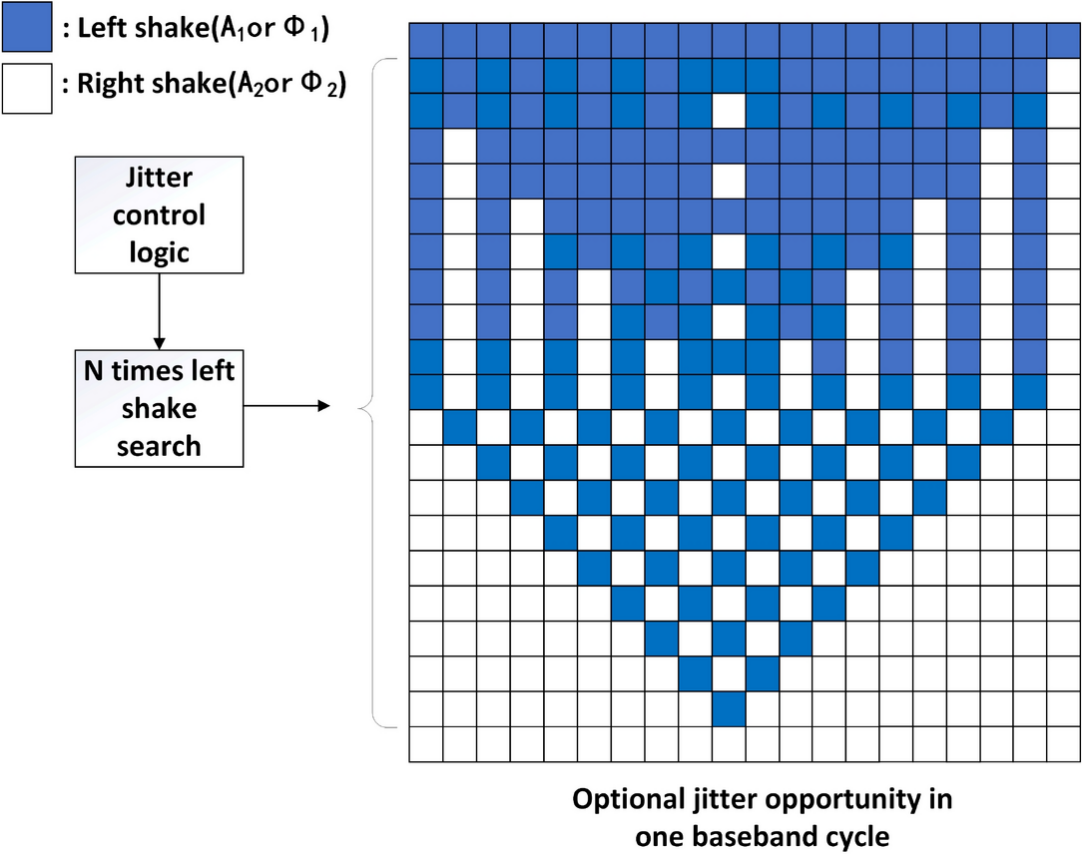
Schematic diagram of jitter LUT.

Next, we will introduce the control scheme of jitter-enhanced PWM modulation. The function of jitter is to improve the resolution after rounding quantization, and the ratio of specific phase and amplitude to left and right jitter depends on the distance between the two adjacent quantization points after shaping filtering. The mapping table of phase jitter and amplitude jitter is stored in the jitter control module. The jitter table with a maximum jitter number of 20 is shown in Fig. 7 . In the table, the left jitter behavior should be arranged in the middle of the baseband period as much as possible, so that the waveform energy of the receiver after baseband filtering can be concentrated in the peak position. In addition to the jitter table shown in Fig. 7, this paper also prepares a jitter table with a maximum jitter number of 5 and 10 to achieve flexible jitter configuration in different baseband cycles and RF cycles.

**Fig. 8:**
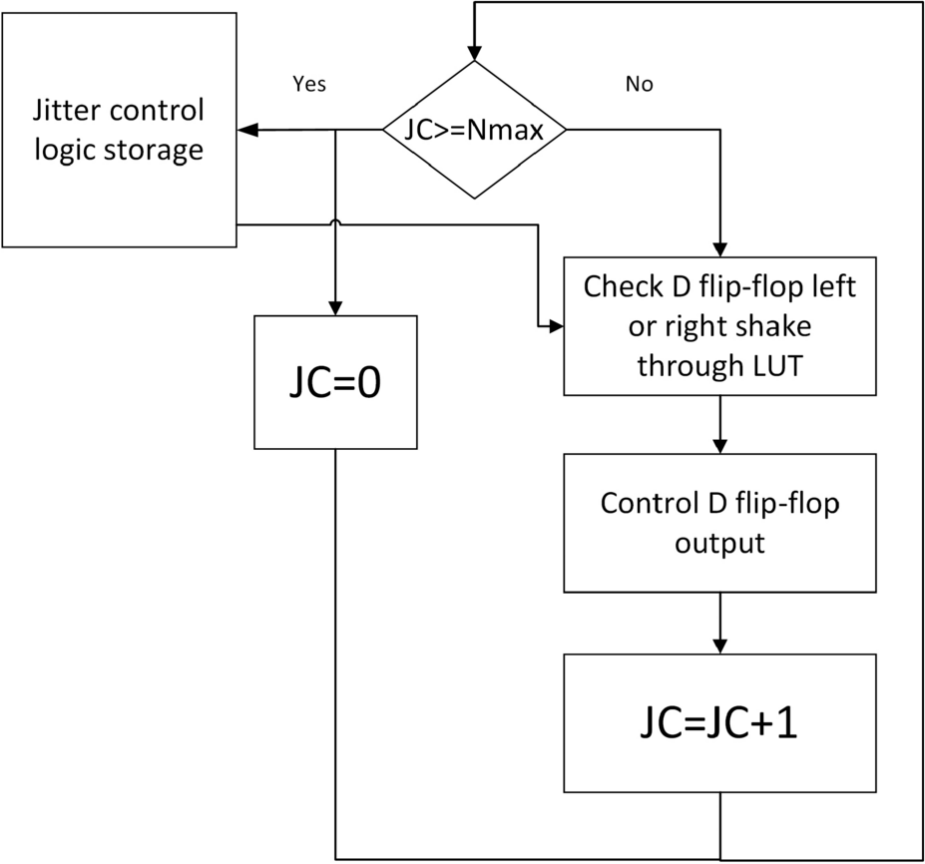
Logic of jitter control module.

The control logic of the jitter control module can be shown in Fig. 8. The input control signal of the jitter control module is the maximum allowable number of jitter and the number of left jitter of phase and amplitude respectively. Through the jitter control signal, the address of the LUT(shown in Fig. 7) corresponding to the jitter configuration is found to control whether the output in the delay line is selected DFF1 or DFF0, corresponding to the left or right jitter behavior. Jitter control after completing a filp-flop selection, the jitter counter (set to JC) is added by 1. A baseband cycle ends when the counter reaches the maximum count Nmax of the radio frequency carrier period within a baseband cycle. At the beginning of the next baseband cycle, the jitter counter is cleared, and the module repeats the above process.

## Simulation result

### Jitter all-digital transmitter launch flow

**Fig. 9:**
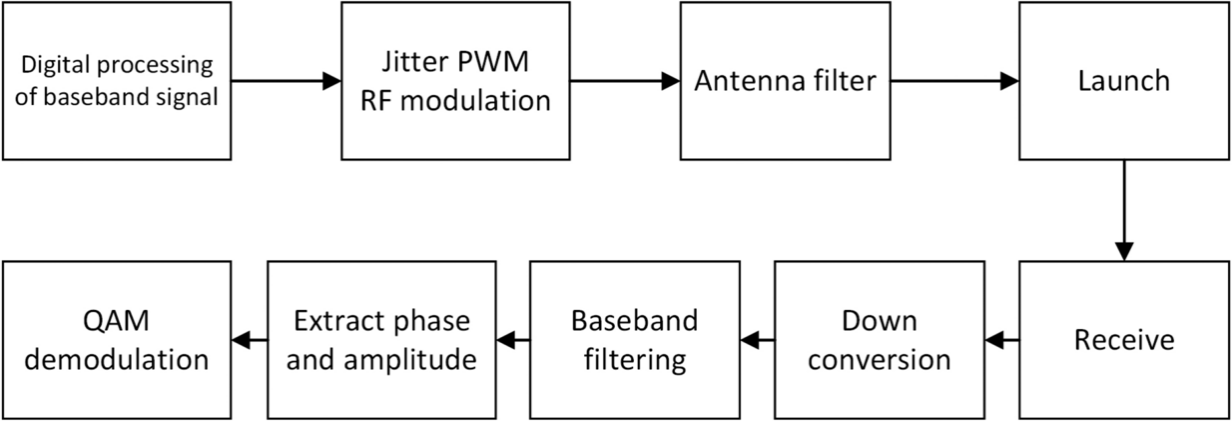
Complete verification process.

The verification system of this paper is to connect the jitter PWM all-digital transmitter proposed directly to the ordinary test receiver. The verification process of jitter PWM all-digital transmitter and receiver is shown in Fig. 9.After the integrated antenna filter, the RF signal wave emitted by the jitter PWM all-digital transmitter in this paper can fully meet the transmission requirements.

**Fig. 10:**
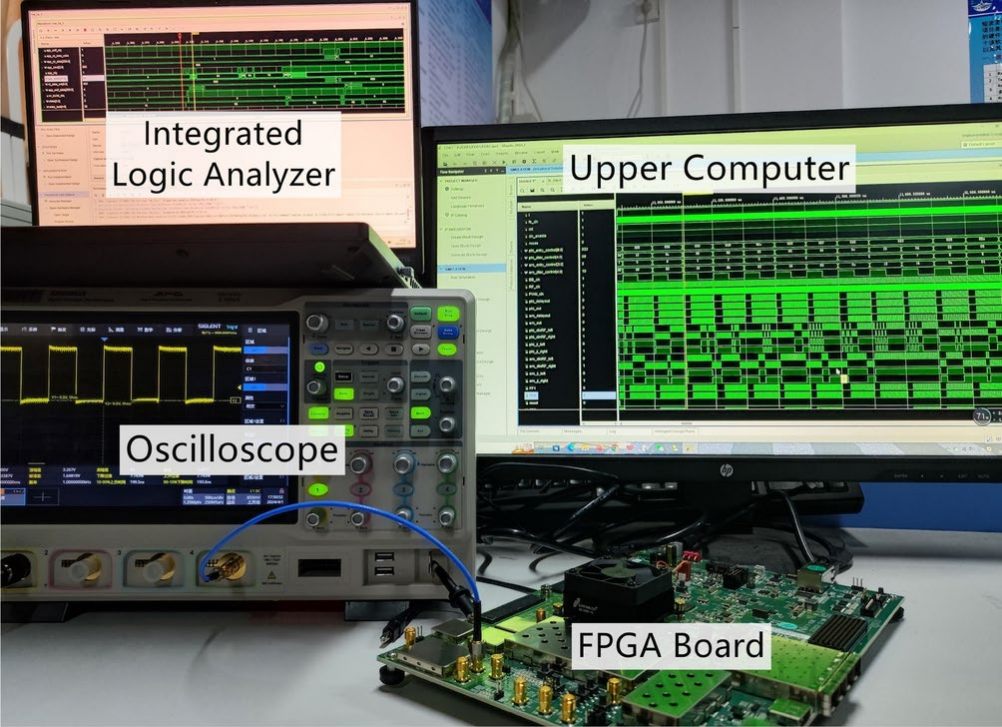
Complete verification process.

Figure 10 shows the test platform based on the Xilinx Zynq UltraScale+ series RFSoC ZCU111 model FPGA board. The platform is mainly composed of ZCU111 FPGA board, upper computer, integrated logic analyzer and oscilloscope. FPGA is responsible for calculating the main body of the data. The upper computer connects the FPGA board through the serial port to obtain the intermediate data in the memory of the FPGA board for further data analysis. The integrated logic analyzer is embedded in Xilinx software “Vivado” and analyze the waveform of the data output together with the oscilloscope. This test platform verifies the digital part of the transmitter architecture proposed in this paper.

**Fig. 11:**
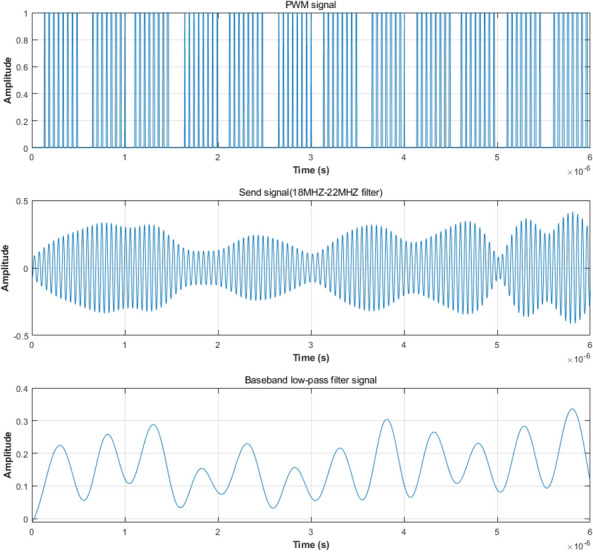
Complete verification process.

**Fig. 12:**
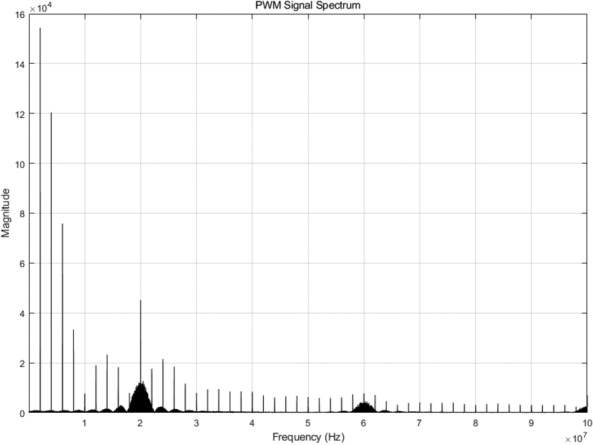
Spectrum of jitter PWM signal.

The process of transmitting and receiving is described in more detail in Fig. 11. The baseband frequency is 2MHZ and the RF frequency is 20MHZ. The jitter PWM signal is sent through an antenna filter of 18MHZ-22MHZ. The total delay of the PWM signal in the baseband period in Fig. 11 corresponds to the baseband phase modulation, and the pulse width corresponds to the RF amplitude modulation. After excluding the DC component, the spectrum of the jitter PWM signal is shown in Fig. 12, which can be used to guide the design of antenna bandpass filters. The send signal shows the waveform of the antenna after filtering out the high-frequency signal, so the final transmitted signal meets the frequency band requirements. The baseband low-pass filter signal in Fig. 11 corresponds to the waveform of the receiver after passing through the baseband filter. We can extract the required amplitude and phase information from the peak and relative position of each waveform to complete the final QAM demodulation.

### Reception performance

The hardware architecture design of this paper is an improvement on the algorithm and system framework of the advanced jitter PWM transmitter. We return the RF signal obtained by this architecture to Matlab software for demodulation. Compared with the original signal, the demodulated signal obtained also achieves the EVM performance improvement that the jitter scheme should bring.

**Table 1:** EVM performance of jitter-enhanced PWM structure for different QAM data at 200 MHz PWM, 20MHZ RF and 1MHZ baseband frequency.

Modulation mode	Whether jitter	RMS EVM (%)	RMS EVM (dB)
QPSK	Yes	0.87	− 41.21
QPSK	No	6.84	− 23.30
16QAM	Yes	0.91	− 40.82
16QAM	No	7.23	− 22.82
64QAM	Yes	0.98	− 40.18
64QAM	No	8.01	− 21.93

**Table 2:** EVM performance of jitter-enhanced PWM structure for different QAM data at 400 MHz PWM, 40MHZ RF and 1MHZ baseband frequency.

Modulation mode	Whether jitter	RMS EVM (%)	RMS EVM (dB)
QPSK	Yes	0.84	− 41.51
QPSK	No	6.69	− 23.49
16QAM	Yes	0.85	− 41.42
16QAM	No	7.04	− 23.05
64QAM	Yes	0.87	− 41.21
64QAM	No	7.78	− 22.18

**Table 3:** EVM performance of jitter-enhanced PWM architecture for 64QAM modulated data at different frequencies by 400MHZ PWM.

Baseband frequency (MHZ)	RF frequency(MHZ)	EVM without jitter(dB)	EVM with jitter(dB)
4	40	− 21.64	− 37.08
4	20	− 21.60	− 36.83
2	40	− 21.83	− 40.18
2	20	− 21.93	− 39.25
2	10	− 22.99	− 38.64
1	40	− 21.18	− 41.21
1	20	− 22.31	− 40.53

**Table 4:** EVM performance of jitter-enhanced PWM architecture for 64QAM modulated data at different frequencies by 200MHZ PWM.

Baseband frequency (MHZ)	RF frequency(MHZ)	EVM without jitter(dB)	EVM with jitter(dB)
2	40	− 21.69	− 37.39
2	20	− 21.64	− 37.08
2	10	− 21.60	− 36.83
1	40	− 21.96	− 40.63
1	20	− 21.93	− 40.18
1	10	− 21.82	− 39.25

**Table 5:** EVM performance of jitter-enhanced PWM architecture for 64QAM modulated data at different frequencies by 150MHZ PWM.

Baseband frequency (MHZ)	RF frequency (MHZ)	EVM without jitter(dB)	EVM with jitter(dB)
1.5	30	− 21.69	− 37.39
1.5	15	− 21.64	− 37.08
1	30	− 21.81	− 40.35
1	15	− 21.76	− 39.66

**Table 6:** EVM performance of jitter-enhanced PWM architecture for 64QAM modulated data at different frequencies by 100MHZ PWM.

Baseband frequency (MHZ)	RF frequency (MHZ)	EVM without jitter(dB)	EVM with jitter(dB)
1	20	− 21.69	− 37.39
1	10	− 21.64	− 37.08
0.5	20	− 21.96	− 40.63
0.5	10	− 21.93	− 40.18

**Table 7:** Hardware cost of jitter-enhanced PWM architecture at different frequencies.

PWM frequency (MHZ)	Baseband frequency (MHZ)	RF frequency (MHZ)	LUT	FF
100	0.5	10	452	231
150	1	15	476	231
200	1	20	485	233
400	1	40	732	468
100/150/200/400	0.5,1/1,1.5/1,2/1,2,4	10,20/15,30/10,20,40/10,20,40	747	472

Table 1 shows the performance comparison of jitter-enhanced PWM architecture with and without jitter-enhanced PWM architecture in the case of 200MHZ PWM,20MHZ RF and 1MHZ baseband frequencies. Table 2 shows comparison between the PWM architecture with and without jitter-enhanced in the case of 400MHZ PWM, 40MHZ RF, and 1MHZ baseband frequencies. It can be seen that the jitter greatly improves the poor demodulation effect without jitter technology. In Tables 3, 4, 5, 6, we give the EVM performance at 0.5, 1, 1.5, 2 MHz baseband frequencies, 10, 15, 20, 30, 40 MHz RF frequencies and 100, 150, 200, 400 MHZ PWM frequencies, respectively. It shows that the higher the ratio of RF frequency to baseband frequency(NRF), the better the EVM effect, which corresponds to the improvement of the effect brought by high jitter times. If NRF is 40, it is equivalent to using jitter to improve the modulation resolution of 5.32bit($log_{2}40$). Because the jitter-enhanced technology is used, the MHZ-level PWM frequency can be used to achieve the same effect as the GHZ-level PWM frequency without jitter. This is a huge promotion and the main contribution of this paper. When the proportions of PWM, RF, and baseband frequencies are the same, the performance remains the same because the same configuration is used in the delay line architecture.

Different baseband and RF frequencies correspond to different control LUT and delay line lengths in the architecture. It is also innovative to use similarity to integrate hardware architectures corresponding to different frequencies. Table 7 shows the hardware cost before and after the integration of architectures. The last row of Table 7 represents the hardware cost required to combine all supported frequencies. In the case of similar hardware architecture, the additional cost of supporting multiple frequencies is only about 2.0% more LUT and 0.8% more FF (filp-flop) than a single frequency. The final design for this paper consumes 0.176% of LUT(425280 available) and 0.055% of register as FF(850560 available). Our design significantly reduces hardware complexity and resource requirements while maintaining reliable operation.

Our power analysis using Vivado shows that the total system power consumption is 1.118 W, of which 1.085 W is static power consumption and 0.033 W is dynamic power consumption. The static power consumption includes Core logic leakage (898 mW) and I/O bank quiescent (187 mW). The main sources of dynamic power consumption are PWM clock power consumption (9.2 mW), RF clock power consumption (2.8 mW), configurable logic block (CLB) power consumption (8 mW), I/O power consumption (8 mW), and signal interconnect power consumption (5 mW). The static power consumption mainly comes from the FPGA core logic and I/O components, while the dynamic power consumption remains relatively low, indicating a high resource utilization rate. This design verifies its low dynamic power consumption characteristics through analysis, providing a reference for optimization and deployment.

In fact, this paper focuses on the design of RF transmission architecture in the shortwave band. Compared with existing architectures, it has significant advantages of less hardware resource occupation, low power consumption, and low complexity. The critical path of this architecture lies in the jitter-enhanced phase delay line between the two clocks(RF_clk and PWM_clk). By optimizing the FSM lookup table logic and reducing the length of the combinational logic, we are now able to achieve RF_clk at 40MHZ and PWM_clk at 400MHZ. Unfortunately, because the design relies on combinational logic implementation, the current architecture has certain limitations in clock frequency, making it difficult to directly expand to higher frequency application scenarios. Due to timing constraints on the critical path, the clock margin of system is gradually exhausted when the RF frequency exceeds 50MHZ. Future work will combine pipeline optimization, critical path improvements, and higher-performance hardware platforms to explore the potential of this approach in higher frequency and larger bandwidth scenarios.

### Comparison and analysis

This section will give a comparison between the all-digital transmitter architecture proposed in this paper and other advanced all-digital transmitters. The performance summary of the architecture proposed in this paper and its comparison with other advanced architectures are shown in Table 8.

**Table 8:** Performance comparison with other advanced architectures.

Reference	fBB (MHZ)	fRF (MHZ)	fPWM (MHZ)	EVM	Technology	Platform
This work	2	10	200	1.44%	Jitter-enhanced PWM	FPGA Xilinx ZCU111
This work	1	40	400	0.87%	Jitter-enhanced PWM	FPGA Xilinx ZCU111
^30^	0.016	1	192	1.70%	Distortion-free PWM	FPGA Xilinx Virtex-7
^30^	6.8	433.3	10,400	5.90%	Distortion-free PWM	FPGA Xilinx Virtex-7
^31^	5	300	19,200	1.00%	Three-level RF-PWM	AWG7122C
^32^	20	200	25,600	0.90%	Outphasing PWM	FPGA Xilinx

Table 8 compares the performance of the architecture proposed in this paper with other state-of-the-art all-digital transmitters. The $f_{RF}$ used in this paper is equivalent to the radio frequency carrier frequency, and the $f_{PWM}$ used refers to the high-speed PWM time resolution frequency. As can be seen from Table 8, the architecture proposed in this paper is slightly improved compared with references^30^ and^31^.For better EVM performance, references^30,31^, and^32^ set the ratio of $f_{PWM}$ to $f_{RF}$ to 192 times, 64 times, and 128 times respectively. In this paper, the lowest ratio of PWM frequency to RF frequency is only 5 times. When traditional transmitters reduce the ratio of PWM frequency to RF frequency, likes references^30^, the EVM will be degraded. The jitter-enhanced PWM technology used in this paper can reduce the requirement of PWM frequency without sacrificing performance, which also means that the frequency requirement of the switching power amplifier of the transmitter can be reduced accordingly. The architecture proposed in this paper achieves both low circuit system complexity and high precision performance.

Although the current design performs excellently in the short-wave frequency band, it faces timing constraints and power consumption limitations in higher frequency applications. The combinational logic of the current architecture (such as lookup table mechanisms and data path selection) increases path delay, limiting the improvement of clock frequency. In order to effectively support operations at higher frequencies, we plan to optimize the critical paths, reduce unnecessary intermediate steps, and simplify the lookup table logic. To reduce system power consumption, we reduce unnecessary signal switching within modules. We also share pipeline modules across several frequency scenarios and optimize the allocation of hardware resources to avoid the use of redundant resources, thereby further reducing system power consumption.

## Conclusion

To improve the efficiency of RF transmitter and minimize energy consumption, this paper proposes an advanced architecture based on jitter to creatively improve the modulation resolution of PWM in time domain, and several frequency schemes are provided. The flexible jitter-enhanced PWM hardware architecture proposed in this paper can achieve excellent EVM performance(a minimum of 0.87% PWM − 41.21dB) while using lower PWM frequencies(400MHz), which helps to reduce the frequency requirements of switching power amplifiers in RF transmitting systems. The choice of this paper is equivalent to a proper tradeoff between low hardware cost and high algorithm performance, which is more suitable for embedded communication systems with strict requirements on efficiency and energy consumption.

The design of this paper has taken into account non-ideal factors and potential implementation challenges in actual systems. In order to simplify circuit logic and reduce timing requirements, a look-up table mechanism is used in the design to ensure the stability of hardware implementation. By selecting a lower PWM frequency, the impact of process deviations and dynamic power consumption is reduced. In the future, we will combine some existing mature technologies to enhance the scalability of the architecture. For example, we will use Multilevel PWM technology to directly improve the modulation resolution of the PWM architecture. This combination can reduce the logic requirements of the jitter-enhanced architecture, meeting the performance needs of modulation resolution while making the architecture better suited for higher frequency scenarios. We will improve system performance through the following optimization efforts: First, by simplifying LUT design and reducing the complexity of combinational logic to speed up the FSM of pipeline and shorten timing path delays; second, by adopting a high-performance FPGA platform, leveraging its more stable clock source and efficient logic resources to further reduce timing delays; and finally, by reasonably allocating hardware resources to optimize the balance between power consumption and performance, ensuring energy efficiency of the system during high-frequency operations.

## Data Availability

The datasets used and/or analysed during the current study available from the corresponding author on reasonable request.
